# Severe Hyperbilirubinemia: A Rare Complication of Lyme Disease

**DOI:** 10.1155/2019/2762389

**Published:** 2019-12-24

**Authors:** Maarij Baig, Lin Zheng, Alka Farmer

**Affiliations:** ^1^Internal Medicine Residency Program, Inspira Medical Center in Vineland, Vineland, NJ 08360, USA; ^2^Division of Hospital Medicine, Department of Medicine, Cooper Medical School of Rowan University, Camden, NJ 08103, USA

## Abstract

Gastrointestinal signs and symptoms are common in the early stages of Lyme disease. However, hyperbilirubinemia from Lyme disease is extremely uncommon. There are only two case reports in literature attributing Lyme disease to hyperbilirubinemia. Here we report a case of severe hyperbilirubinemia as the presenting symptom of Lyme disease. Other plausible etiologies have been ruled out after extensive workups, including liver biopsy. His hyperbilirubinemia gradually resolved after being started on doxycycline. With high incidence of Lyme disease, it should be considered for patients who present with hyperbilirubinemia in endemic areas with possible tick exposure.

## 1. Introduction

Lyme disease is a multisystemic infection caused by the tick-borne spirochete, Borrelia burgdorferi. Gastrointestinal involvement, such as acute hepatitis, could present in the early stages of Lyme disease [1]. However, hyperbilirubinemia secondary to Lyme disease is extremely uncommon. There are only two case reports in literature attributing Lyme disease to hyperbilirubinemia; one of which was in a pediatric patient [2, 3]. Here we present a rare case of hyperbilirubinemia secondary to Lyme disease.

## 2. Case Presentation

A 23-year-old male presented to our hospital with jaundice, fever, and arthralgia in early September. He had been in his usual state of health until approximately six days prior to his presentation. He had experienced diffuse arthralgia and a temperature of 38.9°C. He was also told he had yellowing of his eyes and skin, which prompted his visit to the Emergency Department. The patient had a past history of splenectomy five years prior to presentation resulting from a motor vehicle accident. Most recently, the patient states he had discontinued his intravenous heroin and moved from a tent in a rural town into a group home and restarted Suboxone.

**Figure 1:**
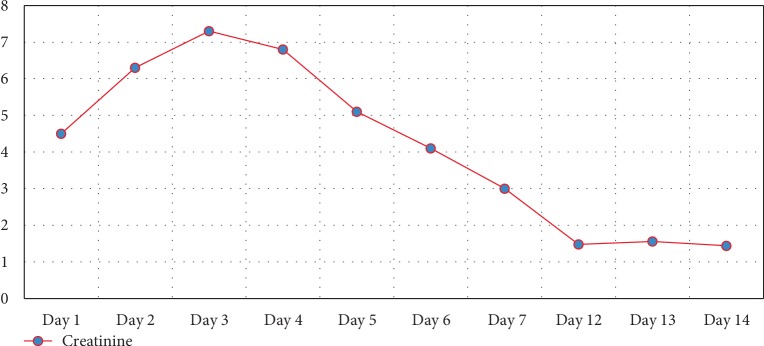
Serum creatine level (in mg/dL) by the hospital day.

**Figure 2:**
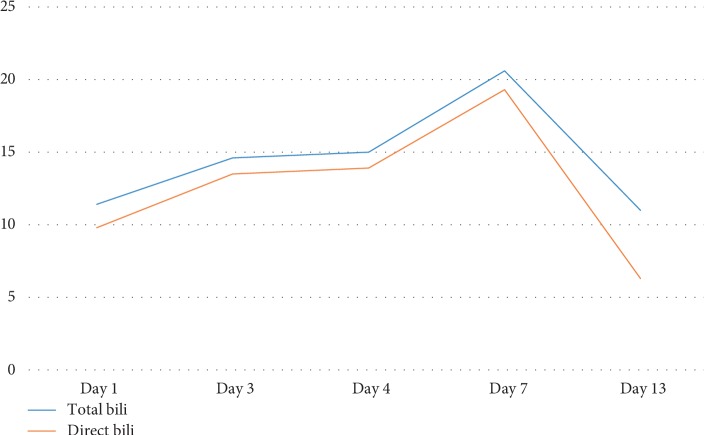
Total and direct bilirubin level (mg/dL) by the hospital day.

**Figure 3:**
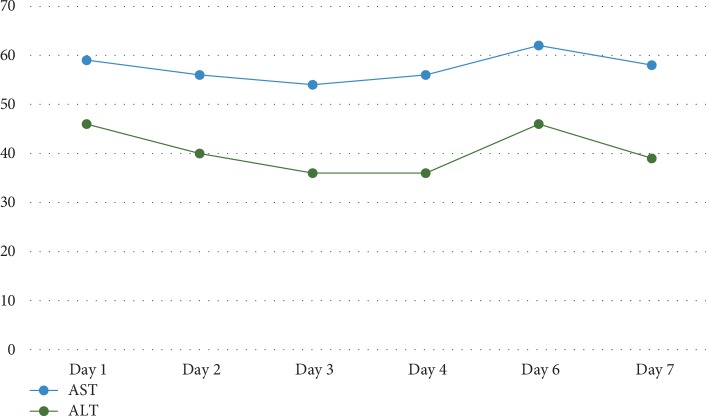
Alanine aminotransferase (ALT, in U/L) and aspartate aminotransferase (AST, in U/L) by the hospital day.

On the day of presentation, initial vitals showed a temperature of 36.7°C, blood pressure of 102/56 mmHg, heart rate of 105 beats per minute, respiratory rate of 20 breaths per minute, and oxygen saturation of 98% on room air. On physical exam, the patient was severely jaundiced with scleral icterus. Examination of the abdomen, joints, skin, and lymph nodes was unremarkable and there were no stigmata of chronic liver disease. His initial laboratory tests showed WBC 25.6 × 10^3^/*µ*L, creatinine of 4.5 mg/dL, total bilirubin 11.4 mg/dL (direct 9.8 mg/dL, indirect 1.6 mg/dL) (Figures 1 and 2), Alanine aminotransferase (ALT) of 46 U/L (Unit/Liter), aspartate aminotransferase (AST) of 59 U/L, and Alkaline Phosphatase (ALK) of 127 U/L (Figure 3).

Abdominal ultrasound was negative, while CT of the thorax, abdomen, and pelvis showed diffuse pulmonary infiltrates, extensive parenchymal changes bilaterally in the lung bases with mediastinal lymph nodes and mesenteric portal hepatic adenopathy. He was started on intravenous piperacillin-tazobactam for possible pulmonary infection.

Extensive workup for conjugated hyperbilirubinemia included viral hepatitis screen, *α* antitrypsin antibody (A1AT), ceruloplasmin, CMV, HIV, antinuclear antibody (ANA), and antismooth muscle antibody, all of which were negative. His LDH was 439 U/L (normal).

There was the concern for tick-borne disease because the patient lived in a tent in the Mid-Atlantic region. Peripheral blood smears were negative for any parasite. Serum screening tests for Lyme disease, Babesiosis, and Ehrlichiosis were ordered. He was started on empirical doxycycline on the third hospital day.

His acute renal injury peaked on hospital day 3 (creatinine 7.3 mg/dL) then gradually trended down. Creatinine improved to 3.0 mg/dL on hospital day 7 (Figure 1). Stool studies, including *Escherichia coli* 0157:H7, were negative.

His total bilirubin continued to rise to 20.6 mg/dL (direct 19.3 mg/dL) on the 7^th^ hospital day (Figure 2), His WBC remained elevated at 20 × 10^3^/*µ*L. However, his AST/ALT remained normal (Figure 3). His International Normalization Ratio (INR) was normal throughout his hospitalization.

The patient remained afebrile and normotensive throughout his stay. Magnetic resonance cholangiopancreatography (MRCP) was subsequently performed and showed no evidence of obstruction or intrahepatic dilatation. Repeat US was performed due to worsening of the laboratory findings and showed heterogeneous echotexture of liver suggesting fatty/fibrotic change. The gallbladder was contracted but no stones were present. Hepatic vessels were patent with appropriate direction of flow. He eventually underwent liver biopsy, which was otherwise negative.

Serum screening tests were predominantly negative except for a positive ELISA screen for Lyme disease, which was subsequently confirmed by Western blot. He was continued on doxycycline. His bilirubin steadily declined. He was discharged home with outpatient follow-up. The patient remained asymptomatic throughout the course of his stay, without abdominal pain or pulmonary symptoms.

## 3. Discussion

Gastrointestinal signs and symptoms are common in the early stages of Lyme disease. One study showed 40% of patients with possible Lyme disease could have abnormal Liver Function Tests (LFTs) [4]. G-glutamyl transpeptidase (GGT) (28%) and alanine transaminase (ALT) (27%) were the most frequently elevated liver function tests among Lyme disease patients [4]. However, hyperbilirubinemia is highly uncommon. Abnormal bilirubin was only seen in 3 out of 115 cases in this study [4]. Similarly, no single case of hyperbilirubinemia was reported in another study, in which a total 314 patients with Lyme disease were included [5]. To the best of our knowledge, this is the first case of hyperbilirubinemia (20 mg/dL) of this extreme attributed to Lyme disease.

Although highly uncommon, Lyme disease should be considered in the differential diagnosis of hyperbilirubinemia, particularly in patients who are at risk of severe infection and end organ damage and are living in an endemic area or have recently travelled to an endemic area, regardless of the presence of rash. Our patient lived in a tent in a wooded area in the Mid-Atlantic region. He presented to the hospital with fever in September. These risk factors should prompt the workup for tick-borne disease.

The combination of ELISA to detect IgM and IgG anti-B. Burgdorferi antibodies and Western blot provides the greatest sensitivity and specificity for the laboratory diagnosis of Lyme disease. Our patient confirmed for *Borrelia burgdorferi* infection. He responded to doxycycline treatment. Extensive workups for other causes of jaundice, including liver biopsy, were negative. He also had multiple end organ damage, including acute renal failure, which could be attributed to Lyme disease associated glomerulonephritis [6].

In conclusion, although highly uncommon, hyperbilirubinemia may be the presenting sign of Lyme disease. Lyme disease should be considered for patients from endemic areas with possible tick exposure.

## References

[1] Zaidi S. A., Singer C. (2002). Gastrointestinal and hepatic manifestations of tickborne diseases in the United States. *Clinical Infectious Diseases*.

[2] Dadamessi I., Brazier F., Smaïl A., Delcenserie R., Dupas J. L., Capron J. P. (2001). Hepatic disorders related to Lyme disease. Study of two cases and a review of the literature. *Gastroentérologie Clinique et Biologique*.

[3] Edwards K. S., Kanengiser S., Li K. I., Glassman M. (1990). Lyme disease presenting as hepatitis and jaundice in a child. *The Pediatric Infectious Disease Journal*.

[4] Horowitz H. W., Dworkin B., Forseter G. (1996). Liver function in early Lyme disease. *Hepatology*.

[5] Steere A. C., Bartenhagen N. H., Craft J. E. (1983). The early clinical manifestations of Lyme disease. *Annals of Internal Medicine*.

[6] Mc Causland F. R., Niedermaier S., Bijol V., Rennke H. G., Choi M. E., Forman J. P. (2011). Lyme disease-associated glomerulonephritis. *Nephrology Dialysis Transplantation*.

